# Identification of regulatory regions of bidirectional genes in cervical cancer

**DOI:** 10.1186/1755-8794-6-S1-S5

**Published:** 2013-01-23

**Authors:** Guohua Wang, Ke Qi, Yuming Zhao, Yu Li, Liran Juan, Mingxiang Teng, Lang Li, Yunlong Liu, Yadong Wang

**Affiliations:** 1 School of Computer Science and Technology, Harbin Institute of Technology, Harbin, Heilongjiang, China; 2 Instrument Science and Technology, Harbin Institute of Technology, Harbin, Heilongjiang, China; 3 School of Life Science and Technology, Harbin Institute of Technology, Harbin, Heilongjiang, China; 4 Information and Computer Engineering College, Northeast Forestry University, Harbin, Heilongjiang, China; 5 Center for Computational Biology and Bioinformatics, Indiana University School of Medicine, Indianapolis, Indiana, USA; 6 Department of Medical and Molecular Genetics, Indiana University School of Medicine, Indianapolis, Indiana, USA

## Abstract

**Background:**

Bidirectional promoters are shared promoter sequences between divergent gene pair (genes proximal to each other on opposite strands), and can regulate the genes in both directions. In the human genome, > 10% of protein-coding genes are arranged head-to-head on opposite strands, with transcription start sites that are separated by < 1,000 base pairs. Many transcription factor binding sites occur in the bidirectional promoters that influence the expression of 2 opposite genes. Recently, RNA polymerase II (RPol II) ChIP-seq data are used to identify the promoters of coding genes and non-coding RNAs. However, a bidirectional promoter with RPol II ChIP-Seq data has not been found.

**Results:**

In some bidirectional promoter regions, the RPol II forms a bi-peak shape, which indicates that 2 promoters are located in the bidirectional region. We have developed a computational approach to identify the regulatory regions of all divergent gene pairs using genome-wide RPol II binding patterns derived from ChIP-seq data, based upon the assumption that the distribution of RPol II binding patterns around the bidirectional promoters are accumulated by RPol II binding of 2 promoters. In HeLa S3 cells, 249 promoter pairs and 1094 single promoters were identified, of which 76 promoters cover only positive genes, 86 promoters cover only negative genes, and 932 promoters cover 2 genes. Gene expression levels and STAT1 binding sites for different promoter categories were therefore examined.

**Conclusions:**

The regulatory region of bidirectional promoter identification based upon RPol II binding patterns provides important temporal and spatial measurements regarding the initiation of transcription. From gene expression and transcription factor binding site analysis, the promoters in bidirectional regions may regulate the closest gene, and STAT1 is involved in primary promoter.

## Background

A major class of adjacently located gene pairs that are divergently transcribed on opposite strands, with < 1000 base pairs separating their transcription start site (TSS) has been identified [1]. These gene pairs are termed "bidirectional" and the regions between the transcription start sites of bidirectional gene pairs, which are also the regulatory region of these genes, are known as bidirectional promoters.

This organization of protein-coding genes is common with > 10% of genes arranged in this configuration [2]. Recent researches have also revealed a large set of previously undiscovered non-coding transcripts near the promoters of protein-coding genes [3]. Compared to adjacently located gene pairs arranged in convergent and tandem configuration, there are more divergent gene pairs with a distance between transcription start sites of < 1000 bp. The percentage of bidirectional gene pairs is significantly larger than expected by chance [2,4].

Bidirectional promoters often regulate DNA repair genes related to cancer and non-DNA housekeeping functions [1,2,5]. Bidirectional promoters are associated with gene pairs whose levels of transcription need to be expressed in a coordinately mechanism, such as genes expressed in different stages of the cell cycle [6], and genes co-expressed in the same biological pathway [7,8]. The bidirectional arrangement of promoters is also highly conserved among different species, which indicates functional importance [2].

Bidirectional promoters are GC rich, with a median GC-content of 66% [2]. There is also a frequent presence of CpG islands near bidirectional promoters. For example, most RNA polymerase II-transcribed genes whose promoters are bidirectional have a CpG island between them [1,9]. The chance of TATA occurrence in bidirectional promoters is significantly lower compared to the genome average, as instead of bidirectional promoters, the motif of TATA sequence is commonly discovered in non-bidirectional promoters [10]. Likewise, the majority of known vertebrate motifs are underrepresented while there is small set of motifs which are overrepresented in bidirectional promoters. More bidirectional gene pairs are transcribed in a given cell than other genes because signals of active transcription, such as occupancy of RNA polymerase II and the modified histones H3K4me2, H3K4me3, and H3ac, are in a higher level around bidirectional promoters [11].

Attempts to identify regulatory regions in the genome involved many experimental and computational methods. One strategy has been to analyze sequence composition, such as GC content, level of conservation, transcription factor binding sites and expressed sequence tags [12-15]. Some strategies are based on the distribution of epigenetic marks that encode regions of transcriptional initiation. For example, by analyzing the ChIP-Chip data of several histone acetylation and methylation markers within the ENCODE region, Heintzman *et al*. found that active promoters could be identified using H3K4me3 profile [16]. With the advance of high resolution sequencing technology, several studies applied histone marker binding sites with ChIP-Seq data to identify promoters [17]. RPol II ChIP-Seq data was also utilized to identify promoters [18-23]. Barski *et al*. [24] showed a clear peak of RPol II level at transcription start site, and found that RPol II binding was positively correlated with gene expression levels [24]. However, no study has identified a bidirectional promoter with RPol II ChIP-Seq data.

We have developed a computational approach to identify the promoter regions of all divergent gene pairs using genome-wide RPol II binding patterns derived from ChIP-seq data, based upon the assumption that the distribution of RPol II binding patterns around the bidirectional promoters are accumulated by RPol II binding of two promoters. We also evaluated the regulatory function of promoters according to the gene expression and STAT1 binding sites.

## Results

### RPol II binding patterns around the bidirectional promoter region

**Figure 1:**
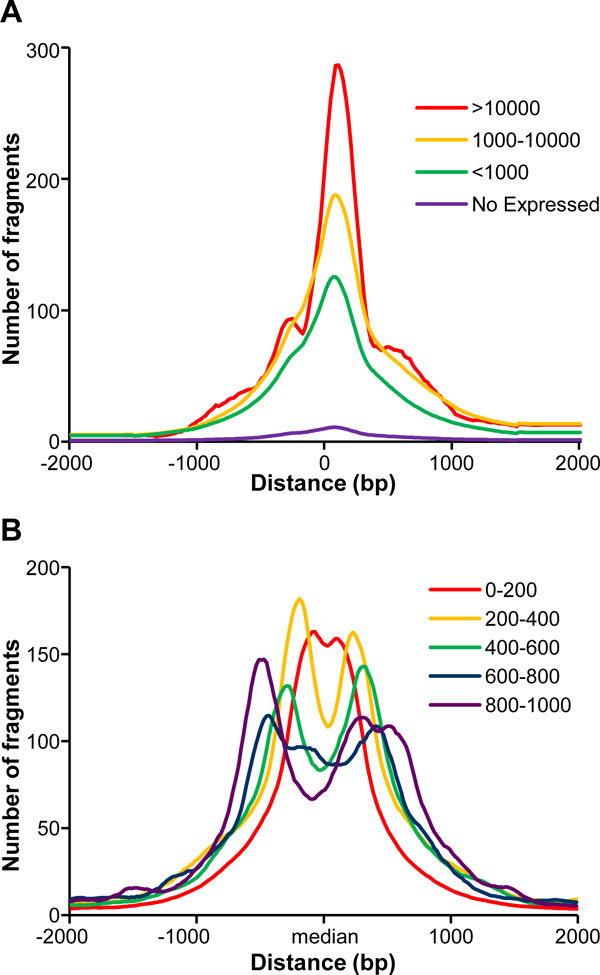
**RPol II binding fragments surrounding TSSs of protein coding genes and bidrectional gene pairs in Hela cells**. (A) ChIP-Seq-derived RPol II binding pattern around the TSS of protein coding gene. Protein-coding genes (n = 6802) whose transcript lengths are > 10,000-bp and no other genes are present within 10,000-bp of their TSS were separated into 4 groups, based upon their expression levels, which were measured using microarray experiments. (B) ChIP-Seq-derived RPol II binding pattern around theTSSs of bidrectional gene pairs. The gene pairs were separated into 5 groups, based on the distance between 2 opposite genes.

We have examined the RPol II binding pattern around the TSS of common protein coding gene and the bidirectional promoter region. To avoid the RPol II binding effect of a gene's neighbourhood, focus was only on the genes whose transcript lengths were > 10,000 bp and no other genes were presented within 10,000 bp of their TSSs. This identified 4,120 expressed genes and 2,682 unexpressed genes in HeLa cells, based on the gene expression array data. We divided the genomic regions into multiple 20 bp bins and calculated the total number of RPol II derived fragments located in each bins within 2,000 bp upstream and downstream of the TSS, producing a RPol II binding landscape in the regulatory regions of the expressed genes. Not unexpectedly, a significant enrichment of the RPol II signal on top of the TSS was seen (Figure 1A), which gradually declined towards both upstream and downstream (transcript) regions. We sub-classified expressed genes based upon their expression levels, and genes with higher expression levels tended to display higher than average RPol II signals around the TSS (Figure 1A). For the coding genes with undetectable (Absent) expression levels, RPol II enrichment around the TSS was markedly lower.

**Figure 2:**
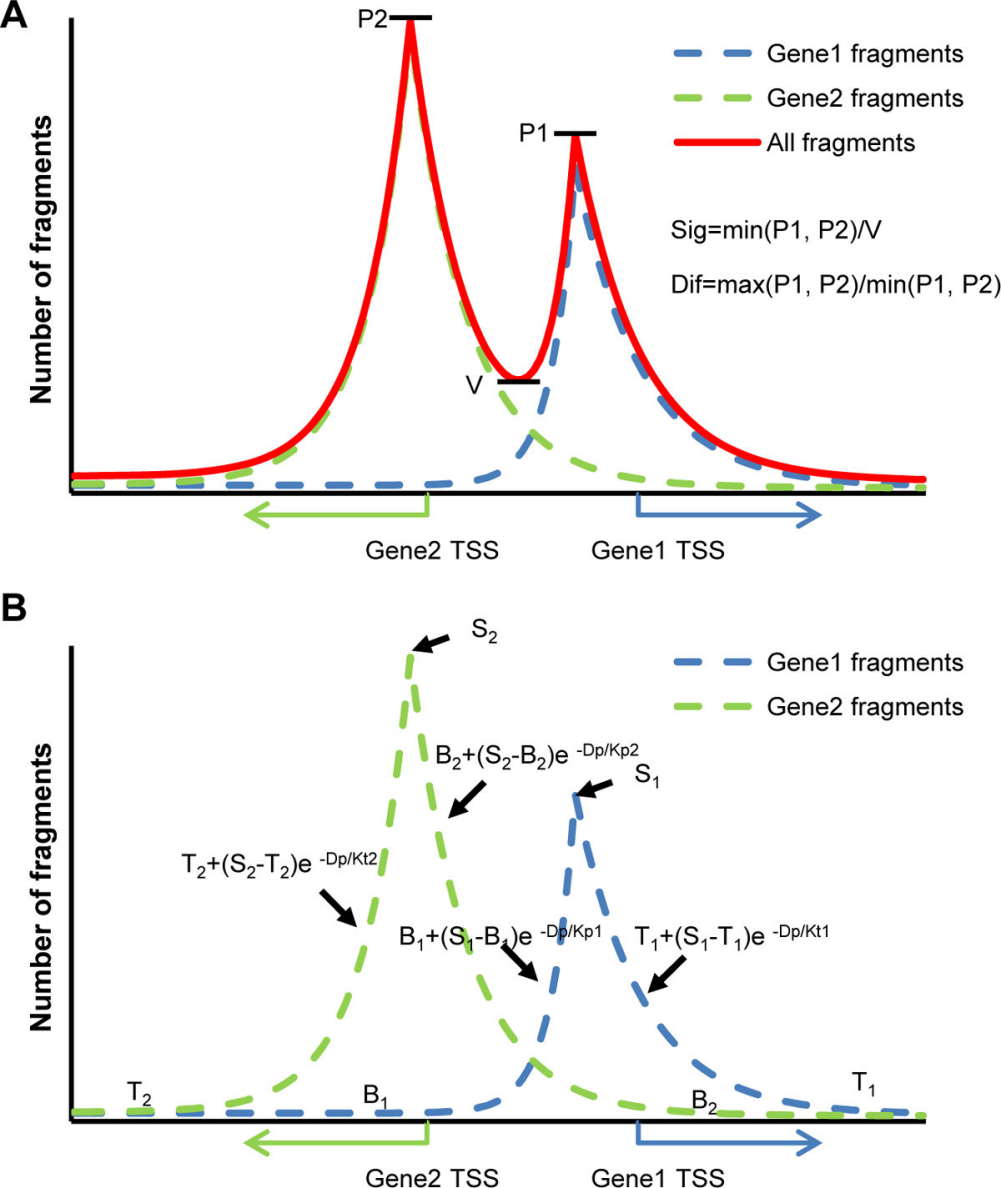
**Statistical model of RPol II distribution of Bi-peak shape**. (A) RPol II binding fragments on 2 promoters form a bi-peak shape. Green and blue dotted lines represent the RPol II distribution surrounding TSSs of 2 opposite genes. The red line presents the accumulation of RPol II fragments. P1, P2, and V are 3 features of the bi-peak shape. The parameters, Sig and Dif, are used to identify the bi-peak shape of RPol II distribution in bidirectional regions.(B) A statistical model of RPol II binding pattern surrounding theTSSs of bi-directional gene pairs. The adjacent genomic regions are divided into multiple 20-bp bins, in which the number of RPol II fragments is assumed to follow a *Poisson *distribution for each promoter. For each of these, the overall binding pattern coud be characterized by 5 hidden variables, including 3 variables describing the expected number of fragments in the background region (B), the transcript region (T), and the bin that contains TSS (S), and 2 variables modeling the signal decay rates in both upstream and downstream of the TSS (K_p _and K_t_). Each hidden variable follows a gamma distribution genome-wide. For the accumulation of RPol II fragments of two promoters, the number of RPol II fragments also follow a *Poisson *distribution.

Removing the same distance between the different isoforms of genes, 1564 head-to-head paired genes in which 522 pairs included the non-coding genes were used to analysis. The numbers of RPol II fragments in each 20 bp bins within 2,000 bp upstream and downstream of the centre of bidirectional regions were calculated. Based on the distance between 2 opposite genes, the bidirectional regions were divided into 5 categories, and the average RPol II signals of each category were calculated (Figure 2B). RPol II signals formed a significant bi-peak shape, and there was a peak-valley in the middle of bidirectional region, which indicated that 2 promoters were located in the bidirectional region. As the distance got shorter, the 2 peaks tended to be close to each other and the higher peak-valley was evident. Within a 200 bp distance, the 2 peaks tended to overlap and form one peak in the middle of the bidirectional region. Therefore, the RPol II signals can be used to identify each regulatory region and the overlap region for 2 promoters of the opposite gene pair.

### Identification of the bi-peak shape in the bidirectional promoter region

In the bidirectional promoter region, RPol II signals usually formed 3 shapes, bi-peak, single peak and no peak. Bi-peak denotes 2 different promoters located in the bidirectional promoter region, and each promoter regulates its closest gene. Single peak had 2 options: (1) the regulatory regions of 2 opposite genes were very close, RPol II bound the same regulatory region; and (2) RPol II bound only one gene promoter, and no RPol II bound the other.

To find the exact regulatory regions, it was important to identify the RPol II bi-peak shape between the TSSs of 2 opposite genes. We adopted 3 features to identify bi-peak distribution of RPol II on bidirectional promoters: P1 - the number of RPol II fragments at TSS of the gene on the positive strand, P2 - the number of RPol II fragments at TSS of the gene on the negative strand, and V - the lowest number of RPol II fragments between the TSSs (Figure 2A). Two parameters, the significant ratio and the difference ratio of RPol II binding pattern between two peaks, were described by these 3 features (see Method). The significant ratio presents the enrichment of RPol II in the lower peak, and the difference ratio presents the difference of RPol II binding between 2 peaks. Larger significant ratio and smaller difference ratio support a strong bi-peak distribution of RPol II fragments.

To estimate the cutoff of significant ratio and difference ratio for bi-peak distribution of RPol II, we simulated some bi-peak shapes using both expressed genes, and single peak shapes with an expressed gene and an unexpressed gene. The paired genes randomly selected from 4,120 expressed genes and 2,682 unexpressed genes were arranged head-to-head, and RPol II signals around the TSSs of genes were summed. According to the distance between paired genes, we simulated 5 sets, each of which included 10,000 bi-peak shapes and 10,000 single peak shapes.

**Figure 3:**
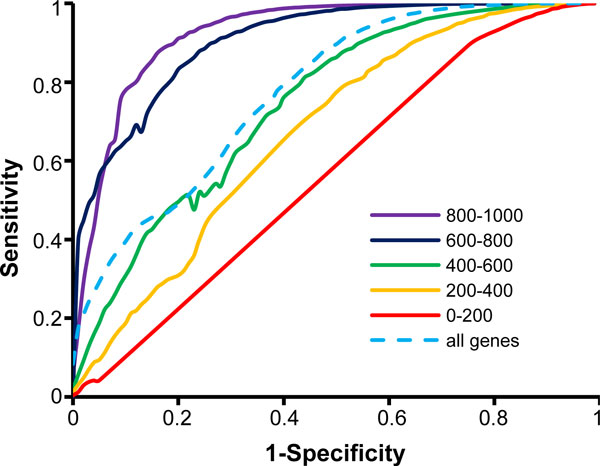
**ROC curve for Bi-peak shape identification**. According to the distance between paired genes, 5 categories of bi-peak shapes and single peak shapes simulated from expressed genes and unexpressed genes are presented in different color. The bi-peak shapes and single peak shapes were considered positive and negative sets, respectively. The ROC curve was generated using ROCR library in the R project http://www.r-project.org.

The area under the curve (AUC) in the Receiver Operator Characteristic (ROC) reached 0.78 in differentiating all bi-peak shapes and single peak shapes of RPol II signals (Figure 3), suggesting an effective distinguished power. Figure 3 clearly shows that the distinguished accuracy of our model is higher for paired genes that have longer distance.

### Identification of regulatory regions in bidirectional promoter

In the regulatory region, the total number of RPol II binding fragments should follow a Poisson distribution; a Poisson mixture model had already been used to identify the microRNA regulatory regions based on the genome wide RPol II binding patterns of protein coding genes [19]. Briefly, the 5 parameters *S, B, T, K_p_*, and *K_t _*determine the Poisson parameter, *λ*, associated with the distribution of the number of RPol II binding fragments. In the bidirectional promoter region that showed bi-peak distribution of RPol II signals, 10 parameters were used to describe RPol II binding pattern of 2 different promoters (Figure 2B). Particle Swarm Optimization (PSO) algorithm was used to maximize probability, and gave the optimized parameters. Genomic regions with < 90% RPol II signal decay compared with those ones in TSS-bin were considered as potential regulatory regions. Two regulatory regions were recognized for each gene pair previously characterized as bi-peak.

**Figure 4:**
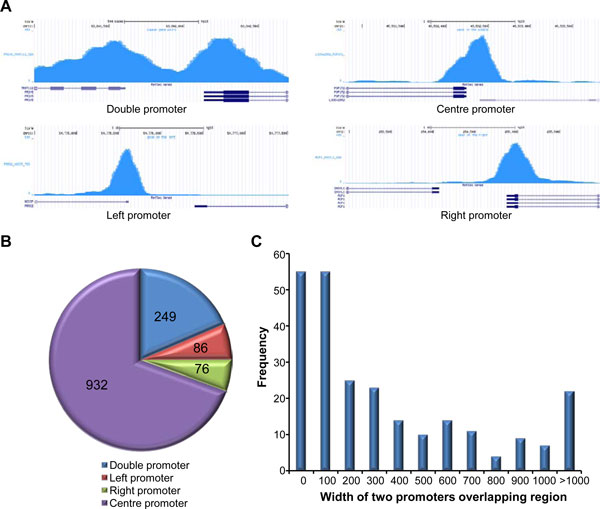
**Identification of promoters in bidirectional regions**. (A) 4 types of promoters. The RPol II binding pattern of 3 types of promoters, double promoter, centre promoter, left promoter and right promoter, showed in UCSC genome browser. (B) The number of identified 4 types of promoter. (C) Histogram illustrating width of overlapping regions of 2 promoters.

The regulatory regions located in the bidirectional region were divided into 4 categories, double promoters which included 2 individual promoters, left promoters that only cover the TSS of gene in negative strand, right promoters that only cover the TSS of gene in right stand, and centre promoters that cover 2 TSSs. Figure 4A shows the RPol II binding pattern of four types of promoters. To assess the efficiency of bi-peak identification, false discovery rate (FDR) was calculated by comparing the number of simulated bi-peaks and single peaks. Using an FDR ≤ 0.2, the significant ratio ≥ 1.2 and difference ratio ≤ 4, 249 bi-peaks (Additional file 1) and 1094 single peaks (Additional file 2) were identified from the bidirectional promoter regions (Figure 4A). After using the *Poisson *mixture model to identify the regulatory region of each gene, the overlaps of regulatory regions of 249 gene pairs are shown in Figure 4B. No overlap of regulatory regions were found for 55 pairs of genes, and the width of overlapped regulatory regions in 135 (54%) gene pairs were < 200 bp (Figure 4C), which indicates that the promoters of these opposite genes are relatively independent. For RPol II signals following the single peak shapes, 86 left promoters, 76 right promoters and 932 centre promoters in Hela cell were identified (Figure 4A).

### Expression of paired genes

**Figure 5:**
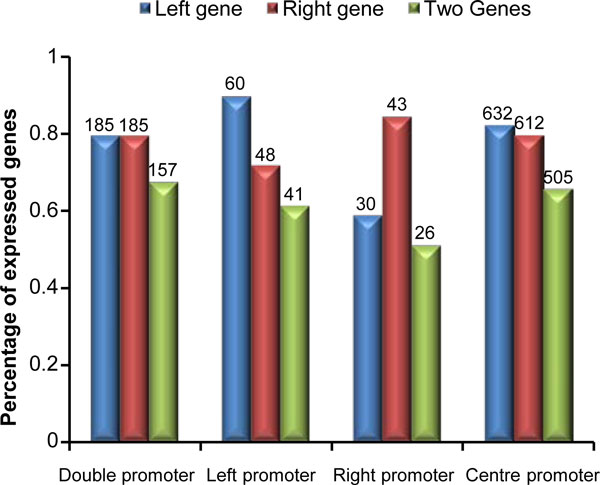
**Percentage of expressed genes associated with 4 types of promoters**. The blue, red and green colors correspond to expressed left genes, right genes and 2 genes, respectively. The numbers on the top of the bars give the total of expressed genes.

Expression levels of each of the paired genes were checked to examine the function of identified regulatory regions. Removing the genes not printed in the expression array, 233 pairs of promoters, 67 left promoters, 51 right promoters, and 770 centre promoters were kept. In total 3 microarrays experiments, genes presented at least twice were expressed, others were not. Figure 5 shows the number and percentage of expressed left gene, right gene, and 2 genes in bidirectional region under the different promoter categories. The percentage of expressed left gene and right gene were similar in double promoter and centre promoter categories, indicating that the regulation of promoters to each strand was unbiased. In the left promoter category, the left gene was presented more than the right one (90 vs 71%), which indicates that left promoters regulate left genes; in contrary, the right promoters tended to regulate the right genes.

### STAT1 binding site in the promoter region

**Figure 6:**
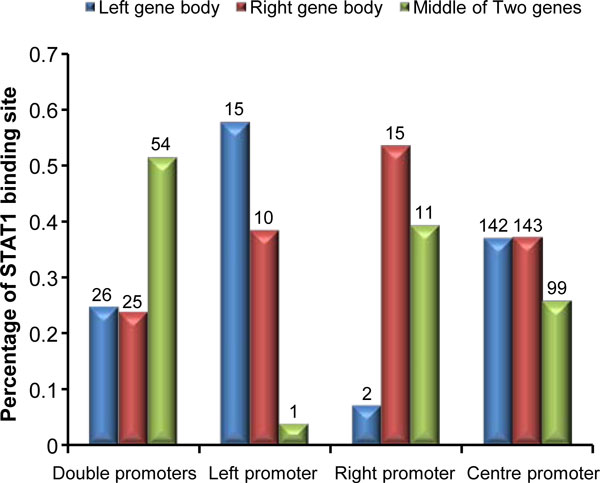
**Percentage of STAT1 binding sites associated with 4 types of promoters**. The blue, red and green colors correspond to STAT1 binding sites located in the left, the right and the middle of 2 genes, respectively. The numbers on the top of bars represent the total STAT1 binding sites.

To see the effect of transcription factor on bidirectional region, overlapping of ChIP-seq-derived STAT1 binding regions, identified by Gerstein's group [11], with the regulatory regions of four types of promoters (Additional file 3, 4, 5 and 6) were explored. To avoid the bias where left promoters and right promoters only cover the TSS of one gene, the regions were extend to 500 bp of the opposite gene body. Figure 6 shows the number of the centre of ChIP-seq-derived STAT1-enriched regions located in left gene body, right gene body, and the middle of two genes in 4 promoter categories. Intuitively, in left promoter category more STAT1 binding sites were located in the left gene body, and same pattern was found in right category, which suggests that STAT1 is involved in the primary promoter.

## Discussion

High throughput RPol II ChIP-seq technology gives the opportunity to identify the promoter region and examine gene regulation. We have reported on a bioinformatics strategy to look at regulatory regions in bidirectional promoter regions, based upon ChIP-seq-derived genome-wide binding patterns of transcription factors and RNA polymerase II; there are 249 double promoters, 76 right promoters, 86 left promoters and 932 centre promoters. Their gene expression and STAT1 binding sites for different promoter categories were followed.

The method of identification of bi-peak shape and single peak is straightforward, and the power of the method is better in the long bidirectional promoter than in the shorter one. Within 200 bp distance, only 25 double promoters (0.03% of 863 total gene pairs) were identified. RPol II signals located around 2 TSSs tend to completely overlap and form one peak in the middle of bidirectional region, making it difficult to distinguish the 2 promoters and single promoter in these regions.

In double promoter and centre promoter categories, > 60% paired genes were expressed. We also found that some genes were expressed, whereas other genes on the other side were unexpressed. This suggests that some other mechanism, such as post-transcriptional regulation, can effect gene expression. The RPol II signal in the gene body or RNA-seq data can be recruited to assess gene regulation by promoters.

Between the genomic region of 2 genes, the proportion of STAT1 binding sites of double promoters was higher than other 3 promoter categories, which indicates that these STAT1 binding sites are important in recruiting RPol II for both genes. Otherwise, to avoid the effect on the other side genes in the left and right promoter categories, more STAT1 binds on the genomics region of gene body. Compared to the gene body region, less STAT1 binding sites were found between the TSSs in the centre promoter category, which suggests that some left promoters and right promoters could not be distinguished from the centre promoter category.

## Methods

### Data description

ChIP-seq data was downloaded from GEO(GSE12783), in which genome-wide binding patterns of transcription factor RPol II and STAT1 in HeLa S3 cells were detected using the GA II platform from Illumina [25]. The reads uniquely mapped to human genome (NCBI build 36) were extended to 200 bp for further analysis. Gene expression data measured with the Affymetrix platform was also downloaded from GEO (GSE3051) [26], and signal intensities were extracted using its Microarray Suite 5.0 (MAS5).

### Features of binding patterns of RPol II in bidirectional promoter

To distinguish bi-peak and single peak binding patterns of RPol II on bidirectional promoters, we introduce features associated with RPol II binding pattern at the TSS. As there is a significant enrichment of RPol II signal here of each protein coding gene, the level of RPol II signal at both TSSs should be noticeably higher than those between them if there is a bi-peak distribution of RPol II on bidirectional promoters. Thus, we define the significance of RPol II binding pattern at TSSs as:

(1) $$Sig\text{ = }\frac{min\left(P1,P2\right)}{V}$$

where P1 is the number of RPol II fragments at TSS of the gene on the positive strand, with P2 being the one on the negative strand. V represents the lowest number of RPol II fragments between the 2 TSSs (Figure 2A). In addition, the difference between the levels of RPol II signal at both TSSs should be smaller than a certain amount if there is a bi-peak. Thus we define the difference of RPol II binding pattern at TSSs as:

(2) $$Dif\text{ = }\frac{max\left(P1,P2\right)}{min\left(P1,P2\right)}$$

We simulate some "bidirectional promoters" using both expressed genes, and others with an expressed and an unexpressed gene. There is a bi-peak RPol II signal on the former promoters and a single peak signal on the latter. We confirmed the amount of the 2 features, a and b, which can distinguish the 2 patterns in the best degree with the simulated data. These 2 features were used to identify bi-peak and single peak patterns on real bidirectional promoters. If Sig > a and Dif < b, the pattern is a bi-peak, otherwise the pattern would be recognized as a single peak.

### Identification of RPol II regulatory regions

The genomic regions neighboring 2 TSSs were divided into 20-bp bins, which were classified into 3 categories: transcript bins of the gene on the negative strand, bins between the 2 TSSs and transcript bins of the gene on the positive strand. For bidirectional promoter region, we assumed the amount of RPol II was the sum of RPol II fragments at the promoters of the 2 genes. The number of RPol II fragments of 2 single promoters detected in each bin should follow a Poisson distribution. The sum of 2 independent Possion distributions also follows Possion distribution, and the parameter λ of the Possion distribution equals the sum of the 2 parameters that belong to the 2 single promoters. The expected RPol II quantity λ_i _of the i-th bin was determined by the following equation.

(3) $$\begin{array}{ccc} \lambda_{i} & ={\left[B_{1}+\left(S_{1}-B_{1}\right)e^{-D_{i1}/K_{p1}}+T_{2}+\left(S_{2}-T_{2}\right)e^{-D_{i2}/K_{t2}}\right]}^{I\left[R_{j}\text{ in NEG}\right]}\; & \\ & \;\;{\left[B_{1}+\left(S_{1}-B_{1}\right)e^{-D_{i1}/K_{p1}}+B_{2}+\left(S_{2}-B_{2}\right)e^{-D_{i2}/K_{p2}}\right]}^{I\left[R_{j}\text{ in MID}\right]}\;\\ & \;\;{\left[T_{1}+\left(S_{1}-T_{1}\right)e^{-D_{i1}/K_{t1}}+B_{2}+\left(S_{2}-B_{2}\right)e^{-D_{i2}/K_{p2}}\right]}^{I\left[R_{j}\text{ in POS}\right]} \end{array}$$

Where S_i_, B_i _and T_i _denote the number of RPol II fragments in the bin of TSS, inter-genetic region and transcription region, respectively (1 represents the parameters associated with the gene on the positive strand, whereas 2 represents those associated with the gene on the negative strand). K_pi _and K_ti _denote the decay rate of RPol II (Figure 2B). D_i1 _denotes the distance between the i-th bin and TSS of the gene on the positive strand, whereas D_i2 _denotes the distance between the i-th bin and TSS of the gene on the negative strand.

A mixture model was established to describe the probability of RPol II fragments neighboring a certain gene pair, where X denotes the observed number of RPol II fragments in each bins, and Y={ B_1_, B_2_, T_1_, T_2_, S_1_, S_2_, Kp_1_, Kp_2_, Kt_1_, Kt_2 _}, each follows a gamma distribution in the genome wide among all the expressed genes. The probability density function is modeled by the following equation:

(4) Pr(X,Y|θ)=Pr(X|Y)•Pr(Y|θ)

(5) $$\text{Pr}\text{(}X\text{|}Y\text{)}\text{ = }\overset{50}{\underset{\begin{array}{c} \text{ i}=1,\\ R_{i}\;in\text{ NEG} \end{array}}{\prod }}\frac{e^{-\lambda_{i}}{\lambda_{i}}^{X_{i}}}{X_{i}!}•\overset{}{\underset{\begin{array}{c} \text{ i}=1,\\ R_{i}\;in\text{ MID} \end{array}}{\prod }}\frac{e^{-\lambda_{i}}{\lambda_{i}}^{X_{i}}}{X_{i}!}•\overset{50}{\underset{\begin{array}{c} \text{ i}=1,\\ R_{i}\;in\text{ POS} \end{array}}{\prod }}\frac{e^{-\lambda_{i}}{\lambda_{i}}^{X_{i}}}{X_{i}!}$$

(6) $$\begin{array}{c} \mathrm{Pr}\left(Y|\theta \right)=\frac{{\left(Kp_{1}Kp_{2}\right)}^{\alpha_{Kp}-1}e^{-\left(Kp_{1}+Kp_{2}\right)/\beta_{Kp}}}{{\left(\Gamma \left(\alpha_{Kp}\right){\beta_{Kp}}^{\alpha_{Kp}}\right)}^{2}}•\frac{{\left(Kt_{1}Kt_{2}\right)}^{\alpha_{Kt}-1}e^{-\left(Kt_{1}+Kt_{2}\right)/\beta_{Kt}}}{{\left(\Gamma \left(\alpha_{Kt}\right){\beta_{Kt}}^{\alpha_{Kt}}\right)}^{2}}\\ •\frac{{\left(B_{1}B_{2}\right)}^{\alpha_{B}-1}e^{-\left(B_{1}+B_{2}\right)/\beta_{B}}}{{\left(\Gamma \left(\alpha_{B}\right){\beta_{B}}^{\alpha_{B}}\right)}^{2}}•\frac{{\left(T_{1}T_{2}\right)}^{\alpha_{T}-1}e^{-\left(T_{1}+T_{2}\right)/\beta_{T}}}{{\left(\Gamma \left(\alpha_{T}\right){\beta_{T}}^{\alpha_{T}}\right)}^{2}}•\frac{{\left(S_{1}S_{2}\right)}^{\alpha_{S}-1}e^{-\left(S_{1}+S_{2}\right)/\beta_{S}}}{{\left(\Gamma \left(\alpha_{S}\right){\beta_{S}}^{\alpha_{S}}\right)}^{2}} \end{array}$$

where the vector θ = {α_B_, β_B_, α_T_, β_T_, α_S_, β_S_, α_Kp_, β_Kp_, α_Kt_, β_Kt_} is the gamma distribution parameters.

Following a similar strategy as previously [19], we used PSO algorithm to estimate the vector θ and maximize the probability of equation (4), and got the optimized parameters Y={ B_1_, B_2_, T_1_, T_2_, S_1_, S_2_, Kp_1_, Kp_2_, Kt_1_, Kt_2 _} as a result. Two regulatory regions were recognized when a certain gene pair was characterized as a bi-peak.

A previous method [19] was used to identify the regulatory regions of single peak gene pairs. If the regulatory region covered one TSS, we considered the region as the promoter of the corresponding gene; where regulatory region covers both TSSs, the promoter corresponds to 2 genes.

## Competing interests

The authors declare that they have no competing interests.

## Authors' contributions

GW, KQ, and YW contributed to the design of the study. GW and KQ designed and performed the computational modeling and drafted the manuscript. YZ, YL, LJ, MT and YW participated in coordination, discussions related to result interpretation and revision of the manuscript. All the authors read and approved the final manuscript.

## Supplementary Material

Additional file 1 **Table of 249 bidirectional gene pairs with bi-peak RPol II signal and their regulatory regions**. Click here for file

Additional file 2 **Table of 1094 bidirectional gene pairs with single peak Rpol II signal and their regulatory regions**. Click here for file

Additional file 3 **Stat1 binding sites of bidirectional promoters with single peak in the middle**. Click here for file

Additional file 4 **Stat1 binding sites of bidirectional promoters with bi-peak**. Click here for file

Additional file 5 **Stat1 binding sites of bidirectional promoters with single peak on the right**. Click here for file

Additional file 6 **Stat1 binding sites of bidirectional promoters with single peak on the left**. Click here for file
